# Conjugative Plasmid-Mediated Extended Spectrum Cephalosporin Resistance in Genetically Diverse *Escherichia coli* from a Chicken Slaughterhouse

**DOI:** 10.3390/ani11092491

**Published:** 2021-08-25

**Authors:** Bai Wei, Ke Shang, Se-Yeoun Cha, Jun-Feng Zhang, Hyung-Kwan Jang, Min Kang

**Affiliations:** 1 Department of Veterinary Infectious Diseases and Avian Diseases, College of Veterinary Medicine and Center for Poultry Diseases Control, Jeonbuk National University, Iksan 54596, Korea; weibai116@hotmail.com (B.W.); shangke0624@gmail.com (K.S.); kshmnk@hanmail.net (S.-Y.C.); jfzhang018@gmail.com (J.-F.Z.); 2 Bio Disease Control (BIOD) Co., Ltd., Iksan 54596, Korea

**Keywords:** extended-spectrum cephalosporins, *E. coli*, chicken, slaughterhouse, genetically diverse, conjugative plasmid

## Abstract

**Simple Summary:**

Extended-spectrum cephalosporin (ESC)-resistant *Enterobacteriaceae* frequently detected in humans and food-producing animals are of major concern in public health. This study was undertaken to investigate the contamination of ESC-resistant *E. coli* in the environment of a slaughterhouse during chicken meat processing. This study indicates that cross-contamination of ESBL/AmpC-producing *E. coli* has a crucial impact on the occurrence of ESC resistance in retail chicken meat. Thus, ESBL-/AmpC-producing *E. coli* were brought into the slaughterhouse by certain broiler chicken flocks, and other chicken flocks were contaminated by ESBL/AmpC-producing *E. coli* already present in the slaughterhouse environment. These findings support the hypothesis that world widely epidemic conjugative plasmids have contributed to the dissemination of ESBL/AmpC resistance in broiler chickens in Korea. As conjugative plasmids always carrying multiple resistance genes, continuous persistence of *bla*_CTX-M_ and *bla*_CMY_ genes located on plasmids within microbial communities will be mediated by co-selection processes with other resistance genes. Hence, further research on the control of bacterial conjugation is urgently required. Our study emphasizes that chicken slaughterhouses could perform the functions of convergence and dispersion of ESBL/AmpC resistance, and that world widely epidemic conjugative plasmids contribute to the dissemination of ESBL/AmpC from chickens to humans along the food chain.

**Abstract:**

ESC-resistant *E. coli* isolates were collected from broiler chickens, a slaughterhouse, and retail meat to assess their dispersion and their involvement in cross-contamination. ESBL-/AmpC-producing *E. coli* were isolated during the slaughter process of all six investigated chicken flocks from scalding, feather removal, first conveyor, evisceration, second washing, third conveyor, and third washing areas, and from handling workers in the slaughterhouse. ESC-resistant *E. coli* isolates with the same pulsed-field gel electrophoresis type were found in the same site (scalding) on different sampling days. ESBL/AmpC-producing *E. coli* isolates were absent in the lairage area in the slaughterhouse, but present in the retail markets in 36.8% (7/19) of the chicken flocks. The *bla*_CTX-M_ genes and *bla*_CMY-2_ were conjugated to recipient *E. coli* J53 in 67.5% (27/40) and 56.1% (23/41) of ESBL-producing and AmpC-producing *E. coli* isolates, respectively. The presence of the same conjugative plasmids was found in genetic diversity ESC-resistant *E. coli* colonies collected on different sampling days. Our study emphasizes that cross-contamination of ESBL/AmpC-producing *E. coli* in slaughterhouse has a crucial impact on the occurrence of ESC resistance in retail chicken meat.

## 1. Introduction

Bacterial resistance, especially to 3rd generation cephalosporins, is of great concern to public health due to the limitations of choice of therapy for human infections. Extended-spectrum cephalosporin (ESC)-resistant *Enterobacteriaceae* are frequently detected in humans and food-producing animals, as well as in the environment [1]. As food-producing animals, especially broiler chickens, are considered possible reservoirs for ESC-resistant *Enterobacteriaceae*, meat and other foodstuffs of animal origin are considered potential sources for the colonization or infection of humans [2].

*Escherichia coli* isolates resistant to ESC mainly due to the acquisition of the resistant genes encoding for extended-spectrum β-lactamases (ESBLs) and plasmid-mediated AmpC (pAmpC) enzymes. The ESBLs consist of several families, and the main enzymes in ESC-resistant *E. coli* are the CTX-M and SHV types, and CMY-type enzymes are the most frequently reported pAmpC β-lactamase [3,4]. Conjugative plasmids carrying ESBL and pAmpC genes are frequently co-harbored genes encoding resistant to other antibiotics, and these conjugative plasmids could maintain and transfer within different bacterial communities. The plasmids harbored ESBL and pAmpC genes have been associated with different transferable replicon types, such as IncA/C and IncI1 [3,4,5]. *E. coli* strains bearing plasmids with ESC resistance, capable of successful conjugative transfer in chicken, could promote the horizontal spread and dissemination to other bacterial hosts, from food-producing animals to humans [6]. This may also play a crucial role in the spread and maintenance of ESC resistance in broiler chicken production.

It has been suggested that there are many stages during the poultry slaughtering process where cross-contamination of foodborne pathogens of *Salmonella* and *Campylobacter* can occur [7,8]. There is, however, only limited evidence available that demonstrates the cross-contamination of ESC-resistant *E. coli* in slaughterhouses [9]. Research has shown that ESC-resistant *E. coli* transmission may occur throughout the whole poultry production chain, and the possible contamination of chicken carcasses with ESC-resistant *E. coli* within the chicken slaughterhouse. This study was undertaken to investigate the ESC-resistant *E. coli* contamination in the slaughterhouse environment along the chicken processing chain. ESC-resistant *E. coli* isolates from broiler chickens, the slaughterhouse environment, and retail meat products, were investigated in order to (i) define the clonal relationships between the isolates for the assessment of the dissemination of the recovered ESC-resistant strains and their involvement in cross-contamination, and (ii) assess the horizontal ESC resistant gene transfer in *E. coli* isolates from broiler chicken slaughterhouses and retail meat by conjugation assays.

## 2. Materials and Methods

### 2.1. Sampling

Twenty-five Korean broiler chicken flocks were investigated for ESC-resistant *E. coli* from November 2015 to October 2016. All chicken flocks were slaughtered at the same slaughterhouse, which is the largest chicken slaughter and processing plant in Korea. The size of the flocks ranged from 50,000 to 100,000 broiler chickens. All 25 flocks were sampled on separate days.

The sampling was performed as follows: firstly, fresh pooled samples of chicken feces were collected from all over the lairage area, and 3–6 pooled samples were collected from each broiler chicken flock. The environmental samples were collected from the first batch on each sampling day at 6 time points from 12 slaughterhouse process sites, including (1) lairage, (2) scalding, (3) feather removal, (4) the first conveyor, (5) evisceration, (6) the first washing, (7) the second conveyor, (8) the second washing, (9) air chilling, (10) the third conveyor, (11) the third washing, and (12) handling workers along the entire poultry processing operation in the slaughterhouse. All samples were collected using sterile cotton gauze, as described previously [8]. All samples were transported to the laboratory on the same day in cooled transport containers with ice for immediate analysis.

Feces samples were collected from an additional 19 broiler chicken flocks from the lairage area and downstream retail chicken meat from the same batch of broiler chicken in the retail market. The raw whole-chicken samples (12–15 samples per flock) were purchased from the retail supermarket and placed on ice in cooled containers and returned to the laboratory for processing within 24 h.

### 2.2. Isolation and Detection

All samples were to investigate the presence of ESC-resistant *E. coli* by selective plating on MacConkey (MC; BD Difco, Sparks, MD, USA) agar supplemented with ceftiofur (8 μg/mL). All colonies on MacConkey agar with different color and morphology were picked and sub-cultured on blood agar plates (Komed, Seongnam, Korea) and confirmed by polymerase chain reaction (PCR) [10]. Three to five typical colonies were collected for each sample. The minimum inhibitory concentrations (MICs) of 16 antimicrobials were determined for confirmed *E. coli* isolates by using the KRNV5F Sensititre™ Broth Microdilution System panel (TREK Diagnostic Systems, Incheon, Korea).

### 2.3. Molecular Characterization

Multiplex PCRs were performed to confirm ESBL/pAmpC resistance genes, and DNA sequence of the resistant genes was performed using an ABI3710 automated sequencer (SolGent, Daejeon, Korea), and sequence comparisons are performed with BLAST (Basic Local Alignment Search Tool). Pulsed-field gel electrophoresis (PFGE) was performed with *Xba*I for all the ESBL/pAmpC-producing isolates, as described previously (https://pulsenetinternational.org, accessed on 20 August 2018).

### 2.4. Conjugation Assay and Molecular Characterization of the Transconjugants

Transfer of ESBL/pAmpC genes to the sodium azide resistant *E. coli* J53 by conjugation was determined by broth-mating experiments [11]. MacConkey agar containing 100 μg/L of sodium azide and 4 μg/mL of ceftiofur was used to select the transconjugants. All the transconjugants were to determine the MICs and the presence of ESBL/pAmpC genes, as described above. Plasmid DNA was extracted from the transconjugants culture using a plasmid Miniprep kit according to the manufacturer’s instructions (Life Technologies, Waltham, MA, USA). The extracted plasmids were analyzed by PCR-based replicon typing [12]. The IncI1 plasmids were further characterized by plasmid multi-locus sequence typing (pMLST) (https://pubmlst.org/plasmid/, accessed on 5 November 2020).

## 3. Results

### 3.1. Distribution of ESBL/AmpC Genes in E. coli Isolates from the Slaughterhouse and Retail Meat

In total, 81 suspected ESBL/AmpC-producing *E. coli* isolates were collected, and all isolates were used to examine the presence of the β-lactamase encoding genes, *bla*_CTX-M_, *bla*_SHV_, and *bla*_TEM_, and the AmpC β-lactamase gene, *bla*_CMY_. Among these 81 isolates, the presence of *bla*_TEM,_ *bla*_CTX-M_, and *bla*_CMY_ was confirmed in 78 isolates (Table 1). The dominant CTX-M types included *bla*_CTX-M-1_ and *bla*_CTX-M-14_, while *bla*_CTX-M-15_, *bla*_CTX-M-55_, and *bla*_CTX-M-65_ were detected in ESBL-producing isolates. Moreover, the only AmpC gene, *bla*_CMY-2_, was found both in the slaughterhouse and retail chicken meat.

Transfer of *bla*_CTX-M_ genes to recipient *E. coli* J53 were found in 67.5% (27/40) of the ESBL-producing isolates. Three *bla*_CTX-M_ genes (*bla*_CTX-M-1_, *bla*_CTX-M-14_, and *bla*_CTX-M-55_) were transferred to J53 in the ESBL-producing *E. coli* isolates from the lairage area, the slaughterhouse environment, and retail meat. The AmpC gene, *bla*_CMY-2_, could be transferred to J53 in 56.1% (23/41) of the AmpC-producing *E. coli* isolates, and its presence was confirmed in AmpC-producing isolates from the lairage area, the slaughterhouse environment, and retail meat.

### 3.2. Occurrence of ESBL/AmpC genes in E. coli Isolates in the Slaughterhouse Environment

The presence of ESBL/pAmpC genes in the *E. coli* isolates in the environment was confirmed during 6 visits on different sampling days to the same slaughterhouse (Table 2). The ESBL/pAmpC genes in *E. coli* were isolated from the scalding, feather removal, first conveyor, evisceration, second washing, third conveyor, and third washing stages, as well as from the handling workers in the slaughterhouse. ESC-resistant *E. coli* were not detected in the first washing, the second conveyor, and air chilling sites in the slaughterhouse.

The clonality of the ESC-resistant *E. coli* isolates from the slaughterhouse typed by *Xba*I-PFGE is shown in Table 2 and Supplementary Figure S1. Clonal diversity of the ESC-resistant *E. coli* isolates was found within and between different sampling days (flocks 1 to 6) in the slaughterhouse environment. However, ESC-resistant *E. coli* isolates with the same PFGE type (type 54) were found in the same site (the scalding area) in the slaughterhouse on different sampling days (flocks 3 and 4).

The conjugative plasmids in ESC-resistant *E. coli* isolates from the slaughterhouse are shown in Table 2. Five types of conjugative plasmids (*bla*_CTX-M-1_ IncI1/ST87, *bla*_TEM-1, CTX-M-55_ IncFIB, *bla*_CMY-2_ IncI1-ST12, *bla*_CMY-2_ IncI1-ST18, and *bla*_CMY-2_ IncI1-ST86) were found in ESC-resistant *E. coli* isolates from the slaughterhouse environment. A comparison of the high genetic diversity of the ESC-resistant *E. coli* colonies from the slaughterhouse environment revealed the presence of the same conjugative plasmids in different ESC-resistant *E. coli* colonies collected on different sampling days.

Of particular interest was the finding that the ESC-resistant *E. coli* isolates from the scalding area of sampling no. 3 and 4 with the same PFGE type 54 contained different conjugative plasmids—*bla*_CMY-2_ IncI1-ST18 and *bla*_CMY-2_ IncI1-ST12. This plasmid, *bla*_CMY-2_ IncI1-ST12, was found in the feather removal and evisceration sites in the slaughterhouse for sampling no. 4. Although ESC-resistant *E. coli* isolates with *bla*_CTX-M-1_ IncI1/ST87 were isolated from the slaughterhouse environment (sampling no. 5, from the feather removal and third conveyor sites), the corresponding flock in the lairage area was negative for ESC-resistant *E. coli*.

### 3.3. Correlation between ESBL/AmpC-Producing E. coli from the Slaughterhouse and Retail Meat

Among the 19 chicken flocks (flocks 7–25) sampled in this study, ESBL/AmpC-producing *E. coli* was detected in 36.8% (7/19) of the chicken flocks (flock 8, 10, 12, 14, 17, 21, and 22). These isolates were found in chicken from the retail markets, but not from the lairage area in the slaughterhouse. Further, 31.6% (6/19) of the ESBL/AmpC-producing *E. coli*-positive chicken flocks did not show the presence of these isolates after processing in the slaughterhouse.

All ESBL/AmpC-producing *E. coli* isolates from the lairage area in the slaughterhouse and the downstream retail meat from the retail market were further analyzed by *Xba*I-PFGE typing and conjugative plasmid typing (Table 3). Most of the ESBL/AmpC-producing *E. coli* isolates from different chicken flocks from the slaughterhouse and retail market showed genetic diversity of PFGE types. *E. coli* isolates with PFGE type 58 were found in different flocks from the lairage area in the slaughterhouse (flock 7) and retail meat (flock 14). *E. coli* isolates from the lairage area in the slaughterhouse with identified PFGE types 10 and 31 were also found in different flocks, namely 9 and 23, and 18 and 20, respectively. Comparing the PFGE genetic diversity of the *E. coli* isolates from the slaughterhouse and retail meat led to the identification of the conjugative plasmids, *bla*_CTX-M-14_ IncI1/ST38, *bla*_CTX-M-14_ IncI1/ST87, and *bla*_CMY-2_ IncI1-ST12, in *E. coli* isolates with different PFGE types from the lairage area and retail meat. Two conjugative plasmids, *bla*_CTX-M-1_ IncI1/ST87 and *bla*_TEM-1, CTX-M-55_ IncN, isolated from retail meat were not found in the lairage area in the slaughterhouse.

## 4. Discussion

Antibiotic resistance is an increasing and evolving phenomenon, with infections due to ESBL/AmpC-producing bacteria being associated with significant morbidity and mortality worldwide [13]. Poultry are the main animal species involved in the spread of these pathogens, and the occurrence of such resistant bacteria in poultry indicates that they may serve as reservoirs of ESC resistance genes, which disseminate to other bacterial species along the food chain [14]. In the present study, the occurrence and distribution of ESBL/AmpC-producing *E. coli* in a slaughterhouse and its downstream retail meat in Korea were investigated. ESBL/AmpC-producing *E. coli* were widely found at various processing stages of chicken production, and certain transmission routes could be confirmed for some foodborne pathogens in chicken during processing in the slaughterhouse [7,8,15]. The environment in fattening farms for broiler chicken can be a source of ESBL/AmpC-producing *E. coli* contamination of retail meat [16]. The detection of ESBL/AmpC-producing *E. coli* in the slaughterhouse environment shows poor control measures for ESBL/AmpC-producing *E. coli* taken during the slaughtering processing, while most hygiene standards and control measures are based on indicative *E. coli* [17].

Cross-contamination in broiler chicken slaughterhouses between flocks is well known [18]. An insufficiently cleaned and disinfected environment may also be a source of poultry flock contamination during processing in the slaughterhouse. The persistence of some isolates in the slaughterhouse environment may constantly contaminate the chicken flocks handled subsequently [8]. In our results, the repeated recovery of ESBL/AmpC-producing *E. coli* isolates with PFGE type 54 from the scalding area in the slaughterhouse on different sampling days showed the potential contamination in the successive chicken flocks by the slaughterhouse environment. Furthermore, some isolates may form biofilms due to their long-term presence in the slaughterhouse environment and cause continuous contamination. This was confirmed by the recovery of the ESBL/AmpC-producing *E. coli* isolate with PFGE type 2 from the third conveyor area in the slaughterhouse (flock 5) and the subsequent recovery from the retail meat of flock no. 22, which was originally ESBL/AmpC-producing-*E. coli*-negative. Therefore, in this scenario, the chicken slaughterhouse performed the functions of convergence and dispersion, with various ESBL/AmpC-producing *E. coli* from chicken flocks from different regions being gathered in the same slaughterhouse and dispersed downstream to the retail meat markets located in different regions. In addition, the ESBL/AmpC-producing-*E. coli*-positive retail meat from ESBL/AmpC-producing-*E. coli*-negative chicken flocks (36.8%, 7/19) in this study emphasizes the role of the slaughterhouse in gathering and spreading the ESBL/AmpC-producing *E. coli* isolates. Furthermore, knowledge of these contamination sources and dissemination modes should be harnessed for the development and application of effective intervention measures against the dissemination of ESBL/AmpC producers during processing in the slaughterhouse.

In addition, slaughterhouses may offer sites for further transfer and dissemination of ESBL/AmpC-resistant genes between various bacterial strains. As shown in our results (Table 2), ESBL/AmpC-producing *E. coli* isolates with PFGE type 54 harboring the conjugative plasmid, *bla*_CMY-2_ IncI1-ST18, were first (sampling date 160412) recovered from the scalding area in the slaughterhouse, while the isolates of PFGE type 54 harboring *bla*_CMY-2_ IncI1-ST12 were recovered from the subsequent sampling at the scalding, feather removal, and evisceration sites. This result suggests the ability of the persistent *E. coli* flora of PFGE type 54 in the slaughterhouse to gain different conjugative plasmids. Studies have confirmed the highly efficient horizontal transfer of resistant plasmids by conjugation in bacterial biofilms and biofilm synthesis by ESBL-producing *E. coli* on numerous surfaces in the environment, including the food chain [19,20,21]. The higher frequency of transfer of the ESBL/AmpC-resistant plasmids in biofilms and the concurrent presence of other antibiotic resistance genes in these plasmids may have serious implications for public health [21]. Thus, it is essential to understand horizontal gene transfer in biofilms and how surface properties in slaughterhouses affect plasmid conjugation, which will aid in controlling resistant plasmid transfer in the future.

Conjugation is the most common way of transferring genetic information and plays a very important role in the spread of multiple antibiotic resistance genes [22]. In this study, 61.7% (50/81) of the ESBL/AmpC-producing *E. coli* isolates showed successful transfer of their ESBL/AmpC resistance by conjugation to another *E. coli* strain. These results suggest that under certain selective pressures, the resistant plasmids were very easily transferred between *E. coli* strains, leading to the spread of ESBL/AmpC resistance, which can be extremely harmful in clinical settings [23]. At several levels of the broiler chicken production chain, *E. coli* isolates harboring conjugative plasmids with *bla*_CMY_ and *bla*_CTX-M_ genes may facilitate horizontal spread and dissemination to other bacterial species in the environment [24]. In addition, *E. coli* strains with conjugative plasmids that persist in the chicken slaughterhouse environment may play an important role in the transmission and maintenance of ESC resistance in the broiler chain, environment, and clinical settings.

Although the IncI1 plasmids—the main type of plasmids found in this study—belong to the narrow-host-range type, IncI1 could successfully spread between different various *Enterobacteriaceae* species and other bacteria in the chicken meat production environment [24]. A noteworthy finding in the present study was that these conjugative plasmids carrying ESBL/AmpC resistance are frequently identified in human clinical isolates, highlighting the potential transfer of these resistant plasmids to humans through the food chain. The most commonly reported AmpC β-lactamase is CMY-2, and plasmid AmpC beta-lactamase CMY-2 is distributed worldwide, particularly in *E*. *coli* and *Salmonella* from food-producing animals and humans [3]. In this study, all the conjugative CMY-2 genes were located on the IncI1 plasmid, which has become one of the most common plasmid families worldwide [25]. The *bla*_CMY-2_ IncI1—belonging to four different STs (ST12, ST18, ST86, and ST108) in our results and the pMLST database—were primarily isolated from *E*. *coli* and *Salmonella* from humans, poultry, and swine in the USA, Canada, UK, and Korea [25]. Notably, the plasmid *bla*_CMY-2_ IncI1-ST12 has been described in several continents, with hosts consisting of both zoonotic pathogens and commensal bacterial species, circulating in both humans and food-producing animals [26,27,28]. This type of plasmid exhibits all the characteristics of a worldwide epidemic plasmid, and the widespread presence of *bla*_CMY-2_ IncI1-ST12 in the chicken meat production chain and the slaughterhouse environment suggests its epidemic potential in Korea in the future [29]. Although the plasmid *bla*_CMY-2_ IncI1 has not been widely reported in ST18, ST86, and ST108, the identification of these conjugative plasmids in clonally diverse *E. coli* isolates may imply its successful dissemination in the chicken meat production chain and retail products in Korea. Therefore, continual surveillance of these plasmids in the broiler chicken meat production chain is essential to reveal the extent of its threat to public health.

IncI1 is recognized as one of the most pervasive plasmids in ESC resistant bacteria found in foods of animal origin [25,30]. Moreover, with *bla*_CTX-M-1_ and *bla*_CTX-M-14_ also being commonly found worldwide, the association between the CTX-M and the IncI1 type of plasmid has been described extensively [24,25,31]. In this study, *bla*_CTX-M-14_-carrying plasmids predominantly belonged to the IncI1 type. These results are contradictory to the findings of a previous study in Korea, which reported the spread of *bla*_CTX-M-14_ in *E. coli* to be mediated mainly by IncF plasmids [32]. It is important to note that *bla*_CTX-M-14_ located on the IncI1 plasmid might have some evolutionary advantage, as it is the only *bla*_CTX-M_ located on the IncI1 plasmid identified in this study. In addition, we found that *bla*_CTX-M-1_ located on the plasmid IncI1 belonged to ST38 and ST87 in our isolates. IncI1 plasmids harboring *bla*_CTX-M-1_ *E. coli*, commonly found in European broiler production, are found in *E. coli* isolates with genetic diversity, indicating that these plasmids successfully disseminate horizontally in the *E. coli* communities spreading from Korean broiler chicken as a source [25]. Our study demonstrates that, due to the dynamic evolution of ESC resistance and horizontal gene transfer of plasmids in food-producing animals, improving the surveillance of ESC resistance would be a key intervention in addressing the rise of resistant bacteria.

## 5. Conclusions

In summary, our results indicate that cross-contamination has a crucial role in the occurrence of ESBL/AmpC-producing *E. coli* in retail chicken meat. It was found that ESBL-/AmpC-producing *E. coli* were brought into the slaughterhouse by certain broiler chicken flocks, and other chicken flocks were contaminated by ESBL/AmpC-producing *E. coli* already present in the slaughterhouse environment. Our study also shows the need not only for intervention measures to prevent contamination with ESBL/AmpC-producing producer on the farm level, but also for effective interventions against cross-contamination with ESBL/AmpC-producing *E. coli* in the slaughterhouse. Therefore, improved cleaning and disinfection measures in the slaughterhouse should be emphasized to avoid further spread of ESBL/AmpC-producing *E. coli*. Our results also support the hypothesis that these world widely epidemic conjugative plasmids have contributed to the dissemination of ESBL/AmpC resistance in broiler chickens in Korea. As conjugative plasmids always carry multiple resistance genes, future persistence of *bla*_CTX-M_ and *bla*_CMY_ genes within the bacterial population will be mediated by co-selection processes with other antimicrobial resistance genes. Hence, further research on the control of bacterial conjugation is urgently required.

## Figures and Tables

**Table 1:** ESC resistance genes in ESC-*E. coli* isolated from the slaughterhouse and retail meat.

Phenotype	No. of Isolates					Conjugation Result (No.)				
	Resistance Genes	Total No.	Slaughterhouse		Retail Meat	Resistance Genes Transfer	Total No.	Slaughterhouse		Retail Meat
			Lairage	Slaughterhouse Environment				Lairage	Slaughterhouse Environment	
ESBL	NA	3	3							
	TEM-1	5	5							
	CTX-M-1	6	5		1	CTX-M-1	6	5		1
	CTX-M-14	7	6		1	CTX-M-14	7	6		1
	CTX-M-15	2	2							
	CTX-M-55	2	1	1						
	CTX-M-65	1	1							
	TEM-1, CTX-M-1	8	4	3	1	CTX-M-1	8	4	3	1
	TEM-1, CTX-M-14	2			2	CTX-M-14	2			2
	TEM-1, CTX-M-55	4		2	2	TEM-1, CTX-M-55	4		2	2
AmpC	CMY-2	24	8	12	4	CMY-2	14	4	8	2
	TEM-1, CMY-2	16	12		4	CMY-2	8	6		2
	TEM-135, CMY-2	1	1			CMY-2	1	1		

NA, not available.

**Table 2:** PFGE types and conjugative plasmid types of ESC-*E. coli* isolates along the slaughter-line in the slaughterhouse.

Flocks No. (Date)	Typing	Slaughterhouse Processing ^a^								
		1. Lairage	2. Scalding	3. Feather Removal	4. First-Convey	5. Evisceration	8. Second-Washing	10. Third-Convey	11. Third-Washing	12. Handling Workers
1 (160405)	PFGE type	11, 20, 29, 37, 49, 56	9				1, 22	22		52
	Conjugative plasmids	NA	NA				NA	*bla*_CMY-2_ IncI1-ST86		*bla*_CMY-2_ IncI1-ST12
2 (160407)	PFGE type	23, 30, 45, 60, 63				2				
	Conjugative plasmids	NA, *bla*_CTX-M-1_IncI1-ST87				*bla*_CMY-2_ IncI1-ST12				
3 (160412)	PFGE type	5, 17	54		40	27				
	Conjugative plasmids	*bla*_CTX-M-1_ IncI1-ST87	*bla*_CMY-2_ IncI1-ST18		*bla*_TEM-1, CTX-M-55_ IncFIB	*bla*_CTX-M-1_ IncI1-ST87				
4 (160414)	PFGE type	6, 19, 35, 48, 51	54	54		54				
	Conjugative plasmids	*bla*_CMY-2_ IncI1-ST12	*bla*_CMY-2_ IncI1-ST12	*bla*_CMY-2_ IncI1-ST12		*bla*_CMY-2_ IncI1-ST12				
		*bla*_CTX-M-1_ IncI1-ST87								
5 (160419)	PFGE type			32			32	25		
	Conjugative plasmids			*bla*_CTX-M-1_ IncI1-ST87			NA	*bla*_CTX-M-1_ IncI1-ST87		
6 (160428)	PFGE type	13, 16						21, 22	33	
	Conjugative plasmids	*bla*_CMY-2_ IncI1-ST108						NA, *bla*_CMY-2_ IncI1-ST12	*bla*_TEM-1, CTX-M-55_ IncFIB	

^a^ The processing of 1 to 12 represents lairage (1), scalding (2), feather removal (3), the first conveyor (4), evisceration (5), the first washing (6), the second conveyor (7), the second washing (8), air chilling (9), the third conveyor (10), the third washing (11), and workers (12). Blank means no isolates; NA means not transferable. ESC-resistant *E. coli* were not detected in the first washing (6), the second conveyor (7), and air chilling (9) in the slaughterhouse.

**Table 3:** PFGE types and conjugative plasmid types of ESC-*E. coli* isolates from slaughterhouse and retail market.

Flock No.	Lairage ^a^		Slaughter-Line		Retail Meat	
	PFGE Type	Conjugative Plasmid	PFGE Type	Conjugative Plasmid	PFGE Type	Conjugative Plasmid
1	11, 20, 29, 37, 49, 56	NA	1, 9, 22, 52	*bla*_CMY-2_ IncI1-ST86	nd	
				*bla*_CMY-2_ IncI1-ST12	nd	
2	23, 30, 45, 60, 63	NA, *bla*_CTX-M-1_ IncI1-ST87	2	*bla*_CMY-2_ IncI1-ST12	nd	
3	5, 17	*bla*_CTX-M-1_ IncI1-ST87	27, 40, 54	*bla*_CMY-2_ IncI1-ST18	nd	
				*bla*_TEM-1, CTX-M-55_ IncFIB	nd	
				*bla*_CTX-M-1_ IncI1-ST87	nd	
4	6, 19, 35, 48, 51	*bla*_CMY-2_ IncI1-ST12	54	*bla*_CMY-2_ IncI1-ST12	nd	
		*bla*_CTX-M-1_ IncI1-ST87			nd	
5	-	-	25, 32	*bla*_CTX-M-1_ IncI1-ST87	nd	
6	13, 16	*bla*_CMY-2_ IncI1-ST108	21, 22, 33	*bla*_CMY-2_ IncI1-ST108	nd	
				*bla*_CMY-2_ IncI1-ST12	nd	
				*bla*_TEM-1, CTX-M-55_ IncFIB	nd	
7	38, 53, 58, 59, 62	*bla*_CMY-2_ IncI1-ST12	nd		-	-
8	-	-	nd		4	*bla*_CTX-M-1_ IncI1-ST87
9	8, 10, 42	*bla*_CTX-M-14_ IncI1-ST162	nd		-	-
10	-	-	nd		36	*bla*_TEM-1, CTX-M-55_ IncN
11	7, 41, 55	NA	nd		nd	nd
12	-	-	nd		34	*bla*_TEM-1, CTX-M-55_ IncN
13	nd	nd	nd		43	*bla*_CTX-M-1_ IncI1-ST87
14	-	-	nd		58	*bla*_CMY-2_ IncI1-ST12
15	3, 15	*bla*_CTX-M-14_ IncI1-ST38	nd		15	*bla*_CMY-2_ IncI1-ST12
16	nd	nd	nd		62	NA
17	-	-	nd		50	*bla*_CTX-M-14_ IncI1-ST87
18	31, 47	*bla*_CMY-2_ IncI1-ST86	nd		-	-
19	26, 57	NA	nd		22, 24	NA
20	18, 31	*bla*_CTX-M-14_ IncI1-ST162	nd		-	-
21	-	-	nd		61	*bla*_CTX-M-14_ IncI1-ST162
22	-	-	nd		25	*bla*_CMY-2_ IncI1-ST12
23	10, 12, 39	NA, *bla*_CTX-M-1_ IncI1-ST38	nd		44	*bla*_CTX-M-14_ IncI1-ST38
24	28	*bla*_CMY-2_ IncI1-ST12	nd		-	-
25	14, 46	*bla*_CTX-M-14_ IncI1-ST87	nd		-	-

- means no ESC-resistant isolates were obtained from the MacConkey agar with ceftiofur; nd means not done; and NA means not transferable.

## Data Availability

The data presented in this study are available from the corresponding author on reasonable request.

## Supplementary Materials

The following are available online at https://www.mdpi.com/article/10.3390/ani11092491/s1, Figure S1. A dendrogram of PFGE profiles for 81 ESBL-/AmpC-producing *E. coli* isolates from the chicken production chain. AMR, antimicrobial resistance; ESC, extended-spectrum cephalosporin; SL, slaughterhouse; Me, retail meat; AMP, ampicillin; AMC, amoxicillin/clavulanic acid; FOX, cefoxitin; FEP, cefepime; TAZ, ceftazidime; XNL, ceftiofur; GEN, gentamicin; STR, streptomycin; TET, tetracycline; CIP, ciprofloxacin; NAL, nalidixic acid; SXT, trimethoprim/sulfamethoxazole; FIS, sulfisoxazole; CHL, chloramphenicol; NA, not available; ST, sequence type.
