# Supplementary material for: Conjugative Plasmid-Mediated Extended Spectrum Cephalosporin Resistance in Genetically Diverse Escherichia coli from a Chicken Slaughterhouse
Source: Animals (Basel). 2021 Aug 25;11(9):2491. doi: 10.3390/ani11092491 (PMC8470599; doi:10.3390/ani11092491)
Supplement: Supplementary file 1 [file animals-11-02491-s001.zip › animals-1316553-supplementary.pdf]

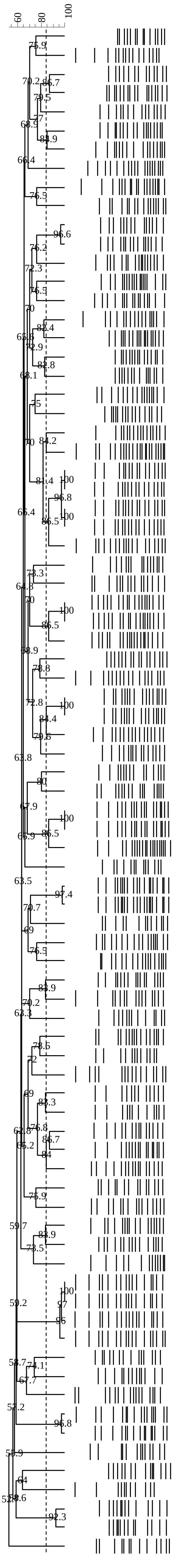

PFGE type	Sampling No.	Source	Location	ESC phenotype	β-lactamase gene	AMR pattern	Transferable *bla* gene-carrying plasmid
1	1	SH	Second-washing	ESBL	CTX-M-55	AMP/FOX/XNL/CAZ/AMC/CIP/NAL/STR/TET/FIS	NA
2	2	SH	Evisceration	AmpC	CMY-2	AMP/XNL/CAZ/FEP/SXT/CIP/NAL/STR/TET/FIS	$bla_{\text{CMY-2}}$ IncI1-ST12
3	15	SH	Lairage	ESBL	CTX-M-1	AMP/FOX/XNL/CAZ/AMC/CIP/NAL/STR/TET	$bla_{\text{CTX-M-1}}$ IncI1-ST38
3	15	RM	Market	AmpC	CMY-2	AMP/XNL/FEP/CIP/NAL/STR/GEN/TET/FIS	$bla_{\text{CMY-2}}$ IncI1-ST12
4	8	RM	Market	ESBL	CTX-M-1	AMP/FOX/XNL/CAZ/AMC/SXT/CIP/NAL/STR/TET/FIS	$bla_{\text{CTX-M-1}}$ IncI1-ST87
5	3	SH	Lairage	ESBL	CTX-M-1	AMP/FOX/XNL/CAZ/AMC/CIP/NAL/STR/TET/FIS	$bla_{\text{CTX-M-1}}$ IncI1-ST87
6	4	SH	Lairage	AmpC	CMY-2	AMP/XNL/CAZ/FEP/CIP/NAL/FIS	$bla_{\text{CMY-2}}$ IncI1-ST12
7	11	SH	Lairage	ESBL	TEM-1	AMP/XNL/CIP/NAL/FIS	NA
8	9	SH	Lairage	ESBL	CTX-M-14	AMP/XNL/FEP/SXT/CIP/NAL/STR/TET/FIS	$bla_{\text{CTX-M-14}}$ IncI1-ST162
9	1	SH	Scalding	AmpC	CMY-2	AMP/FOX/XNL/CAZ/AMC/CIP/NAL/STR/TET/FIS	NA
10	9	SH	Lairage	ESBL	CTX-M-14	AMP/FOX/XNL/CAZ/AMC/CIP/NAL/STR/TET/FIS	$bla_{\text{CTX-M-14}}$ IncI1-ST162
10	23	SH	Lairage	ESBL	CTX-M-15	AMP/XNL/CAZ/NAL/STR/TET/FIS	NA
11	1	SH	Lairage	ESBL	TEM-1	AMP/FOX/XNL/CAZ/AMC/NAL/STR/GEN/TET/FIS	NA
12	23	SH	Lairage	ESBL	TEM-1, CTX-M-1	AMP/XNL/CIP/NAL/TET/FIS	$bla_{\text{CTX-M-1}}$ IncI1-ST87
13	6	SH	Lairage	AmpC	CMY-2	AMP/FOX/XNL/SXT/CIP/NAL/STR/GEN/TET	$bla_{\text{CMY-2}}$ IncI1-ST108
14	25	SH	Lairage	ESBL	CTX-M-14	AMP/FOX/XNL/AMC/CIP/NAL	$bla_{\text{CTX-M-14}}$ IncI1-ST87
15	15	SH	Lairage	ESBL	CTX-M-1	AMP/FOX/XNL/CAZ/AMC/CIP/NAL/STR/TET	$bla_{\text{CTX-M-1}}$ IncI1-ST38
16	6	SH	Lairage	AmpC	CMY-2	AMP/FOX/XNL/CAZ/AMC/NAL/STR/GEN/TET/FIS	$bla_{\text{CMY-2}}$ IncI1-ST108
17	3	SH	Lairage	ESBL	CTX-M-1	AMP/FOX/XNL/CAZ/AMC/CIP/NAL/STR/TET	$bla_{\text{CTX-M-1}}$ IncI1-ST87
18	20	SH	Lairage	ESBL	CTX-M-14	AMP/XNL/CIP/NAL/GEN/TET/FIS	$bla_{\text{CTX-M-14}}$ IncI1-ST162
19	4	SH	Lairage	AmpC	TEM-1, CMY-2	AMP/FOX/XNL/AMC/CIP/NAL/STR/TET	NA
20	1	SH	Lairage	AmpC	TEM-1, CMY-2	AMP/FOX/XNL/CAZ/AMC/CIP/NAL/STR/TET	NA
21	6	SH	Third-convey	AmpC	CMY-2	AMP/FOX/XNL/CAZ/AMC/SXT/CIP/NAL/GEN/TET/FIS	NA
22	1	SH	Second-washing	AmpC	CMY-2	AMP/FOX/XNL/CAZ/AMC/CIP/NAL/STR/FIS	NA
22	1	SH	Third-convey	AmpC	CMY-2	AMP/FOX/XNL/CAZ/AMC/SXT/CIP/NAL/STR	$bla_{\text{CMY-2}}$ IncI1-ST86
22	6	SH	Third-convey	AmpC	CMY-2	AMP/XNL/CAZ/FEP/SXT/CIP/NAL	$bla_{\text{CMY-2}}$ IncI1-ST12
22	19	RM	Market	AmpC	CMY-2	AMP/XNL/CAZ/FEP/NAL/STR/TET/FIS	NA
22	19	RM	Market	AmpC	TEM-1, CMY-2	AMP/FOX/XNL/CAZ/AMC/CIP/NAL	NA
23	2	SH	Lairage	AmpC	CMY-2	AMP/FOX/XNL/CIP/NAL/STR/TET	NA
24	19	RM	Market	AmpC	TEM-1, CMY-2	AMP/FOX/XNL/AMC/CIP/NAL/TET	NA
25	5	SH	Third-convey	ESBL	TEM-1, CTX-M-1	AMP/FOX/XNL/CAZ/AMC/CIP/NAL	$bla_{\text{CTX-M-1}}$ IncI1-ST87
25	22	RM	Market	AmpC	TEM-1, CMY-2	AMP/FOX/XNL/CAZ/AMC/NAL	$bla_{\text{CMY-2}}$ IncI1-ST12
25	22	RM	Market	AmpC	TEM-1, CMY-2	AMP/FOX/XNL/SXT/CIP/NAL/STR/GEN/TET/FIS	$bla_{\text{CMY-2}}$ IncI1-ST12
26	19	SH	Lairage	AmpC	CMY-2	AMP/FOX/XNL/AMC/CIP/NAL/STR/TET	NA
27	3	SH	Evisceration	ESBL	TEM-1, CTX-M-1	AMP/FOX/XNL/CAZ/AMC/CIP/NAL	$bla_{\text{CTX-M-1}}$ IncI1-ST87
28	24	SH	Lairage	AmpC	TEM-1, CMY-2	AMP/FOX/XNL/CAZ/FEP/AMC/CIP/NAL/STR/TET/FIS	$bla_{\text{CMY-2}}$ IncI1-ST12
28	24	SH	Lairage	AmpC	TEM-1, CMY-2	AMP/XNL/SXT/CIP/NAL/FIS	$bla_{\text{CMY-2}}$ IncI1-ST12
29	1	SH	Lairage	ESBL	TEM-1	AMP/FOX/XNL/CAZ/AMC/SXT/CIP/NAL/STR/TET	NA
30	2	SH	Lairage	ESBL	CTX-M-65	AMP/FOX/XNL/AMC/SXT/NAL/STR/TET	NA
31	20	SH	Lairage	ESBL	CTX-M-14	AMP/FOX/XNL/CAZ/AMC/SXT/CIP/NAL/TET	$bla_{\text{CTX-M-14}}$ IncI1-ST162
31	18	SH	Lairage	AmpC	TEM-135, CMY-2	AMP/FOX/XNL/CAZ/AMC/SXT/CIP/NAL/STR/TET/FIS	$bla_{\text{CMY-2}}$ IncI1-ST86
32	5	SH	Second-washing	AmpC	CMY-2	AMP/FOX/XNL/CAZ/AMC/CIP/NAL/STR/TET/FIS	NA
32	5	SH	Feather removal	ESBL	TEM-1, CTX-M-1	AMP/XNL/CIP/NAL/STR/TET/FIS	$bla_{\text{CTX-M-1}}$ IncI1-ST87
33	6	SH	Third-washing	ESBL	TEM-1, CTX-M-55	AMP/XNL/SXT/NAL/STR/GEN/TET/FIS	$bla_{\text{TEM-1, CTX-M-55}}$ IncFIB
34	12	RM	Market	ESBL	TEM-1, CTX-M-55	AMP/XNL/SXT/CIP/NAL/STR/GEN/TET/FIS	$bla_{\text{TEM-1, CTX-M-55}}$ IncN
35	4	SH	Lairage	AmpC	TEM-1, CMY-2	AMP/XNL/CAZ/FEP/SXT/CIP/NAL/STR/TET/FIS	$bla_{\text{CMY-2}}$ IncI1-ST12
35	4	SH	Lairage	ESBL	NA	AMP/FOX/XNL/CAZ/AMC/SXT/CIP/NAL/STR/TET/FIS	NA
36	10	RM	Market	ESBL	TEM-1, CTX-M-55	AMP/XNL/CAZ/FEP/CIP/NAL/TET	$bla_{\text{TEM-1, CTX-M-55}}$ IncN
37	1	SH	Lairage	AmpC	TEM-1, CMY-2	AMP/XNL/NAL/TET/FIS	NA
38	7	SH	Lairage	AmpC	TEM-1, CMY-2	AMP/XNL/FEP/SXT/CIP/NAL/TET/FIS	$bla_{\text{CMY-2}}$ IncI1-ST12
39	23	SH	Lairage	ESBL	CTX-M-1	AMP/FOX/XNL/CAZ/AMC/SXT/CIP/NAL/STR/GEN/TET/FIS	$bla_{\text{CTX-M-1}}$ IncI1-ST38
40	3	SH	First-convey	ESBL	TEM-1, CTX-M-55	AMP/FOX/XNL/CAZ/AMC/CIP/NAL	$bla_{\text{TEM-1, CTX-M-55}}$ IncFIB
41	11	SH	Lairage	AmpC	TEM-1, CMY-2	AMP/XNL/SXT/CIP/NAL/STR/GEN/TET/FIS	NA
42	9	SH	Lairage	ESBL	NA	AMP/FOX/XNL/AMC/CIP/NAL	NA
43	13	RM	Market	ESBL	TEM-1, CTX-M-1	AMP/XNL/CIP/NAL/STR/TET/FIS	$bla_{\text{CTX-M-1}}$ IncI1-ST87
44	23	RM	Market	ESBL	TEM-1, CTX-M-14	AMP/XNL/SXT/CIP/NAL/STR/GEN/TET/FIS	$bla_{\text{CTX-M-14}}$ IncI1-ST38
45	2	SH	Lairage	ESBL	CTX-M-55	AMP/XNL/CAZ/FEP/CIP/NAL/STR/TET	NA
46	25	SH	Lairage	ESBL	CTX-M-14	AMP/XNL/SXT/CIP/NAL/STR/GEN/FIS	$bla_{\text{CTX-M-14}}$ IncI1-ST87
47	18	SH	Lairage	AmpC	TEM-1, CMY-2	AMP/XNL/FEP/CIP/NAL/STR/TET/FIS	$bla_{\text{CMY-2}}$ IncI1-ST86
47	18	SH	Lairage	ESBL	CTX-M-15	AMP/XNL/CIP/NAL/STR/TET/FIS	NA
48	4	SH	Lairage	ESBL	TEM-1, CTX-M-1	AMP/FOX/XNL/CAZ/AMC/CIP/NAL	$bla_{\text{CTX-M-1}}$ IncI1-ST87
49	1	SH	Lairage	ESBL	TEM-1	AMP/FOX/XNL/CAZ/AMC/CIP/NAL/STR	NA
50	17	RM	Market	ESBL	CTX-M-14	AMP/XNL/CIP/NAL/GEN/FIS	$bla_{\text{CTX-M-14}}$ IncI1-ST87
51	4	SH	Lairage	AmpC	TEM-1, CMY-2	AMP/FOX/XNL/CAZ/AMC/NAL/STR/TET/FIS	NA
52	1	SH	Handling workers	AmpC	CMY-2	AMP/FOX/XNL/CAZ/AMC/SXT/CIP/NAL	$bla_{\text{CMY-2}}$ IncI1-ST12
53	7	SH	Lairage	AmpC	TEM-1, CMY-2	AMP/FOX/XNL/CAZ/AMC/CIP/NAL/STR/GEN/TET	$bla_{\text{CMY-2}}$ IncI1-ST12
54	3	SH	Scalding	AmpC	CMY-2	AMP/XNL/FEP/CIP/NAL/TET/FIS	$bla_{\text{CMY-2}}$ IncI1-ST18
54	4	SH	Evisceration	AmpC	CMY-2	AMP/FOX/XNL/CAZ/AMC/CIP/NAL/TET	$bla_{\text{CMY-2}}$ IncI1-ST12
54	4	SH	Feather removal	AmpC	CMY-2	AMP/XNL/FEP/SXT/CIP/NAL/STR/GEN/TET/FIS	$bla_{\text{CMY-2}}$ IncI1-ST12
54	4	SH	Scalding	AmpC	CMY-2	AMP/FOX/XNL/CAZ/AMC/CIP/NAL	$bla_{\text{CMY-2}}$ IncI1-ST12
55	11	SH	Lairage	AmpC	TEM-1, CMY-2	AMP/XNL/SXT/CIP/NAL/STR/TET/FIS	NA
56	1	SH	Lairage	ESBL	TEM-1	AMP/XNL/FEP/CIP/NAL/STR/TET/FIS	NA
57	19	SH	Lairage	ESBL	NA	AMP/XNL/CAZ/FEP/SXT/CIP/NAL/TET	NA
58	14	RM	Market	AmpC	CMY-2	AMP/FOX/XNL/CAZ/AMC/SXT/CIP/NAL/GEN/TET/FIS	$bla_{\text{CMY-2}}$ IncI1-ST12
58	7	SH	Lairage	AmpC	CMY-2	AMP/XNL/SXT/CIP/NAL/STR/GEN/TET/FIS	$bla_{\text{CMY-2}}$ IncI1-ST12
59	7	SH	Lairage	AmpC	CMY-2	AMP/FOX/XNL/CAZ/AMC/SXT/CIP/NAL/STR/GEN/TET	NA
60	2	SH	Lairage	ESBL	TEM-1, CTX-M-1	AMP/XNL/CAZ/FEP/CIP/NAL/STR	$bla_{\text{CTX-M-1}}$ IncI1-ST87
61	21	RM	Market	ESBL	TEM-1, CTX-M-14	AMP/FOX/XNL/CAZ/FEP/CIP/NAL/STR/TET/FIS	$bla_{\text{CTX-M-14}}$ IncI1-ST162
62	16	RM	Market	AmpC	CMY-2	AMP/FOX/XNL/CAZ/AMC/CIP/NAL	NA
62	7	SH	Lairage	AmpC	CMY-2	AMP/XNL/FEP/SXT/CIP/NAL/STR/GEN/TET/FIS	NA
63	2	SH	Lairage	ESBL	TEM-1, CTX-M-1	AMP/FOX/XNL/CAZ/CIP/NAL/GEN/TET	$bla_{\text{CTX-M-1}}$ IncI1-ST87
